# Automatic choroidal segmentation in OCT images using supervised deep learning methods

**DOI:** 10.1038/s41598-019-49816-4

**Published:** 2019-09-16

**Authors:** Jason Kugelman, David Alonso-Caneiro, Scott A. Read, Jared Hamwood, Stephen J. Vincent, Fred K. Chen, Michael J. Collins

**Affiliations:** 1 Contact Lens and Visual Optics Laboratory, School of Optometry and Vision Science, Queensland University of Technology, Brisbane, Queensland Australia; 2 Centre for Ophthalmology and Visual Science, The University of Western Australia, Perth, Australia; 3 Ocular Tissue Engineering Laboratory, Lions Eye Institute, Perth, Australia; 4 Department of Ophthalmology, Royal Perth Hospital, Perth, Australia

**Keywords:** Tomography, Biomedical engineering

## Abstract

The analysis of the choroid in the eye is crucial for our understanding of a range of ocular diseases and physiological processes. Optical coherence tomography (OCT) imaging provides the ability to capture highly detailed cross-sectional images of the choroid yet only a very limited number of commercial OCT instruments provide methods for automatic segmentation of choroidal tissue. Manual annotation of the choroidal boundaries is often performed but this is impractical due to the lengthy time taken to analyse large volumes of images. Therefore, there is a pressing need for reliable and accurate methods to automatically segment choroidal tissue boundaries in OCT images. In this work, a variety of patch-based and fully-convolutional deep learning methods are proposed to accurately determine the location of the choroidal boundaries of interest. The effect of network architecture, patch-size and contrast enhancement methods was tested to better understand the optimal architecture and approach to maximize performance. The results are compared with manual boundary segmentation used as a ground-truth, as well as with a standard image analysis technique. Results of total retinal layer segmentation are also presented for comparison purposes. The findings presented here demonstrate the benefit of deep learning methods for segmentation of the chorio-retinal boundary analysis in OCT images.

## Introduction

The choroid is a vascular tissue layer lining the posterior eye situated between the retina and the sclera. This structure plays a critical role in normal visual, developmental and metabolic function. The provision of nutrients and oxygen to the outer retina, the absorption of stray light, the regulation of ocular temperature and intraocular pressure, and contributions to the processes regulating ocular growth and refractive error development are some of the important functions of the choroid^1^. The choroid is also thought to play an important role in the pathogenesis of a range of sight threatening ocular diseases^2^. Due to its location posterior to the retinal pigment epithelium (RPE), traditional imaging methods (e.g. retinal photography) cannot reliably visualise the choroid. However, in recent decades, the development of optical coherence tomography^3^ (OCT) has allowed the choroid to be imaged and measured *in-vivo*^4^. The analysis of chorio-retinal OCT images has resulted in improved understanding of ocular tissue changes in a range of different conditions including: normal eye development^5,6^, aging^7,8^, refractive errors^9,10^ and eye diseases^11–15^. Therefore, the ability to easily obtain reliable automatic segmentation information from OCT images of the choroid is critical both clinically and for advancing our understanding of the eye through research.

Previous analysis approaches for OCT retinal segmentation have utilised methods based on standard image processing techniques^16,17^. However, with the increasing popularity and advancement in the realm of machine learning, such methods have evolved to include a range of new techniques including support vector machine^18,19^, convolutional neural network (CNN) classifier^20^, random forest classifier^21^, U-net-based fully-convolutional architecture^22–24^ and other deep learning methods^25–32^. Using a method combining a CNN and a graph search (CNN-GS), Fang *et al*.^20^ automatically segmented nine retinal layer boundaries using a patch-based classification approach. Here, small square patches (33 × 33 pixels) are constructed from the full-size OCT images and used to train the CNN. At the evaluation step, patches for every pixel in a test image are classified with the resulting probability map for each boundary used to construct a graph. Finally, the graph search, originally proposed by Chiu *et al*.^33^, outputs the predicted boundary location. Hamwood *et al*.^30^ examined the effect of changing the patch size and network architecture and subsequently improved the performance as a result. Replacing the CNN with an RNN, Kugelman *et al*.^31^ showed that a similar RNN-based approach (RNN-GS) performs competitively to a CNN one.

Similar to retinal segmentation, early methods of choroidal segmentation relied on standard image processing methods^34–41^. However, in contrast to OCT retinal layer segmentation, previous work utilising machine learning methods for choroidal segmentation has been limited. Sui *et al*.^42^ proposed a multi-scale CNN to learn the edge weights in a graph-based approach. Here, the CNN was composed of a coarse-scale, mid-scale and fine-scale network each to learn a different set of features within the images. The output edge costs from the network were used within a graph search to delineate two choroidal boundaries (Bruch’s membrane (BM) and the choroid-scleral interface (CSI)). In a similar approach, Chen *et al*.^43^ used a fully-convolutional encoder-decoder architecture based on SegNet^44^ to output edge probability maps for BM and the CSI. From here, seam carving was used to delineate the boundaries within an image by finding a path of connected pixels across the width of the image. Al-Bander *et al*.^45^ combined superpixel clustering, image enhancement and deep learning to segment the choroid. Here, superpixel-centred patches were classified using a CNN as either choroid or non-choroid, from which the contours defining the edges of the choroid are then resolved. Devalla *et al*.^24^ presented their Dilated-Residual U-Net (DRUNET) architecture to segment the various regions in OCT images including the retina, choroid and optic nerve head. Here, they combined the benefits of skip connections, residual connections and dilated convolutions by incorporating each into their network. Alonso-Caneiro *et al*.^32^ extended the previously proposed patch-based approach for retinal segmentation to additionally segment the choroid-scleral interface in OCT images.

In this paper, a range of deep learning methods for OCT choroidal boundary segmentation are explored. Similar methods have been applied for retinal segmentation in the past, however the application of machine learning methods to choroidal segmentation is significantly less prevalent. This study extends upon our previous work on patch-based approaches to choroidal segmentation^32^ and expands on the use of semantic segmentation architectures. Additionally, there is also limited work investigating the effect of network architecture changes and image pre-processing on the performance of a semantic segmentation approach to this problem. Here, the aim is to investigate the effect of changes in the patch size, network architecture, and image pre-processing as well as the method used (patch-based vs semantic segmentation). For each, the impact on performance was primarily evaluated by comparing the segmentation performance on the chorio-scleral interface (CSI). Given the vast range of machine learning model architectures and associated parameters, this work takes an important step towards understanding the optimal architecture and approach for choroidal boundary segmentation in OCT images. For comparison purposes the segmentation of the total retinal thickness was also evaluated. The outcomes of the approaches presented here are likely to aid in the future for the design and evaluation of machine learning-based OCT image analysis techniques.

## Methods

### OCT data

**Figure 1:**
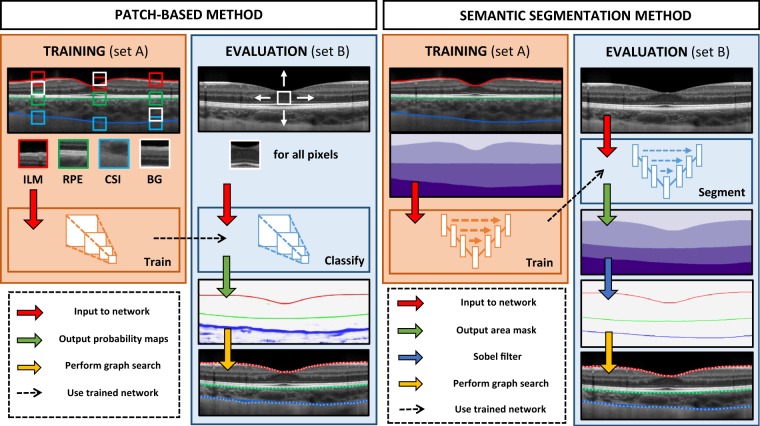
Illustration of the steps involved in each of the two deep learning methods (patch-based and fully-convolutional) proposed in this work for segmentation of the retina and choroid. Where applicable the ILM is marked in red, the RPE in green and the CSI in blue.

The dataset used consists of spectral domain OCT (SD-OCT) scans from a longitudinal study that has been described in detail in a number of previous publications^5,6^. In this study, OCT scans were collected from 101 children at four different visits over an 18-month period. Approval from the Queensland University of Technology human research ethics committee was obtained before the study, and written informed consent was provided by all participating children and their parents. All participants were treated in accordance with the tenets of the Declaration of Helsinki. At each visit, two sets of six foveal centred radial chorio-retinal scans were taken on each subject, however, only the data from the first visit is used in this paper. The scans were acquired using the Heidelberg Spectralis (Heidelberg Engineering, Heidelberg, Germany) SD-OCT instrument using the enhanced depth imaging mode. To improve the signal to noise ratio, automatic real time tracking was used with 30 frames averaged for each scan. The acquired images each measure 1536 × 496 pixels (width x height). With a vertical scale of 3.9 µm per pixel and a horizontal scale of 5.7 µm per pixel which corresponds to an approximate physical area of 8.8 × 1.9 mm. These images were exported as bmp (lossless) images with other related data stored in an accompanying xml file, and subsequently analysed using custom software where an automated graph based method was used to segment three layer boundaries for each image. This segmented data was then assessed by an expert human observer who manually corrected any segmentation errors. The three layer boundaries within the labelled data include the outer boundary of the retinal pigment epithelium (RPE), the inner boundary of the inner limiting membrane (ILM), and the CSI. An example of the positions of these boundaries is shown in Fig. 1.

For computational reasons, only a subset of the dataset described above is utilised here. This consists of a single set of scans (six scans) for 99 participants from their first visit only. These participants are randomly divided into two sets; set A for neural network training and validation (50 participants, 300 B-scans in total) and set B for evaluation (49 participants, 294 B-scans in total). Within set A, an 80/20 split is used for training (40 participants, 240 B-scans) and validation (10 participants, 60 B-scans) with participants selected randomly for each. There is no overlap of participants between the training and validation sets or between sets A and B. Henceforth; ‘A-scan’ refers to a single-column of an OCT image while ‘B-scan’ refers to a full-size OCT image.

### Overview

The deep learning automatic segmentation methods considered in this work are comprised of two main types: patch-based and semantic segmentation. Each method involves a number of steps. Firstly, a set of OCT scans (set A) is used to train a neural network for patch classification (patch-based method) or for area segmentation on full-size B-scans (semantic segmentation method). Next, a second set of OCT scans is used to evaluate the network (set B). For each scan in set B, per-boundary probability maps are constructed by classifying each pixel in the scan (patch-based method) or segmenting the scan and then applying the Sobel filter (semantic segmentation method). In both cases, each probability map is then used to construct a graph, and a boundary position prediction is obtained by performing a shortest-path graph search. The following sections provide greater detail of the two methods while Fig. 1 illustrates the various steps involved in each. Some of the patch based methods have been presented elsewhere^32^. The software environment used throughout this work consists of Keras 2.2.4^46^ using Tensorflow^47^ (GPU) 1.8.0 backend in Python 3.6.4. For the purposes of evaluating the speed of each method an identical hardware and software setup is used. Here, the hardware consists of an Intel Xeon W-2125 CPU, Nvidia GeForce GTX 1080Ti GPU, Samsung SM961 SSD and 32GB 2400 MHz DDR4 ECC RAM.

### Patch-based networks

#### Convolutional neural network (CNN) architecture

Convolutional neural networks (CNNs) have had considerable use and demonstrated success for a range of image classification^48^, and segmentation tasks^49^. CNNs consist of a number of different layers with a set of parameters associated with each layer. Convolutional layers take a number of equal sized kernels (filters) which are convolved with the input and stacked together to produce an output. The parameters include: the kernel size (height × width), the stride lengths (vertical, horizontal), the quantity of zero-padding (top, bottom, left, right) applied to the input, and the number of kernels. Pooling layers takes a single window sliding step-by-step over the input. At each step, an operation is performed to pool the input to a smaller size. Such operations that are commonly used include max pooling (where the maximum value is taken from within the window), and average pooling (where the average of the values is taken). The parameters of this layer include: the window size (height × width), the stride (step) lengths (vertical, horizontal), the quantity of zero-padding applied to the input (top, bottom, left, right) and the pooling operation (max or average). Activation layers are used to introduce non-linearity into neural networks where the rectified linear unit (ReLU)^50^ is a common choice for CNNs and has been shown to outperform other variants such as tanh and sigmoid^51^. Fully-connected (FC) layers are equivalent to convolutional layers where the kernel size is equal to the spatial size of the input and there is no zero-padding applied to the input. Two CNNs with a variety of different patch sizes and complexity are used within this work with the architectures listed in Supplementary Table S1. These include: the Cifar CNN (CNN 1) introduced by Fang *et al*.^20^, and the Complex CNN (CNN 2) presented by Hamwood *et al*.^30^, with variants for a range of patch sizes. Dropout for regularisation has not been used for the CNNs in this work, consistent with previous approaches^20,30^.

#### Recurrent neural network (RNN) architecture

Recurrent neural networks (RNNs) have been widely applied to, and have shown to be useful for, problems involving sequential data such as speech recognition^52,53^, and handwriting recognition^54^. However, there are just a handful of examples of their application to images. To perform OCT image classification using a recurrent neural network, the architecture to be used here is that introduced by Kugelman *et al*.^31^. This network, partially inspired by the ReNet architecture^55^, possesses a number of parameters associated with each layer including: the direction of operation (vertical or horizontal), number of passes (1: unidirectional, 2: bidirectional), number of filters, dropout percentage and receptive field size (height, width). The size of the receptive field represents the size of the region of the input which is processed by the RNN at each step. The direction of operation corresponds to whether the RNN will process each row of a column (vertical) or each column of a row (horizontal) before moving to the next column or row respectively. A unidirectional layer will pass over the input only in a single direction (left to right or top to bottom) whereas a bidirectional layer will additionally pass over the input in the opposite direction (right to left or bottom to top) with the outputs for each pass concatenated along the feature axis. The number of filters in each layer indicates the depth of the output, with the addition of more filters enabling the network to learn an increased number of patterns from the input. The dropout percentage^56^ corresponds to the number of units within a layer that are randomly turned off at each epoch. The RNN architecture used within this work is described in Supplementary Table S2.

#### Training

The Cifar CNN, Complex CNN and RNN networks are trained to perform classification using specific sized (height × width pixels) patches of the OCT images. Here, each patch is assigned to a class based on the layer boundary that it is centred upon, with classes constructed for each of the three layer boundaries of interest (ILM, RPE and CSI) as well as an additional background class (BG) for patches that are not centred upon any of the three layer boundaries. This is a similar procedure to that used in previous work^20,30^. In their work, Fang *et al*.^20^, utilised 33 × 33 patches while Hamwood *et al*.^30^, extended upon this and, using 33 × 33 and 65 × 65 patch sizes, showed that utilising a larger patch size can improve performance. Kugelman *et al*.^31^ also experimented with the patch size using 32 × 32 and 64 × 64 patch sizes as well as 64 × 32 and 32 × 64 sized rectangular patches. Of their tested sizes, the vertically oriented patch size (64 × 32) provided the best trade-off between accuracy and complexity in the context of retinal segmentation using RNNs. With this in mind, to assess the effect on choroidal segmentation, patches of various sizes including 32 × 32, 64 × 32, 64 × 64 and 128 × 32 (height × width pixels) are utilised with layer boundaries centred one pixel above and to the left of the central point.

Patches are constructed for training (~1,200,000 patches) and validation (~300,000 patches) from the data in set A. In each scan, three boundary patches and one random background patch are sampled from each column ensuring equally balanced classes. However, patches are only created within a cropped region of each scan (approximately 100 pixels from the left to 250 pixels from the right) due to the lack of true boundary locations present as a result of the optic nerve head as well as shadowing within this region for some scans. The Adam algorithm^57^ with default parameters ($\alpha =0.001,\,{\beta }_{1}=0.9,\,{\beta }_{2}=0.999,\,{\epsilon }=1\times {10}^{-8})$ is used for training to minimise cross-entropy loss with each network trained until convergence is observed with respect to the validation loss. No early-stopping is employed. Here, convergence is determined based on the inspection of the validation losses. No transfer learning is performed. Instead, each network is trained from scratch with weights initialised using small random values. Afterwards, the model with the highest validation accuracy (percentage of patches correctly classified) is chosen for evaluation. It should also be noted that no learning rate schedule is used.

### Semantic segmentation networks

#### Architecture

**Figure 2:**
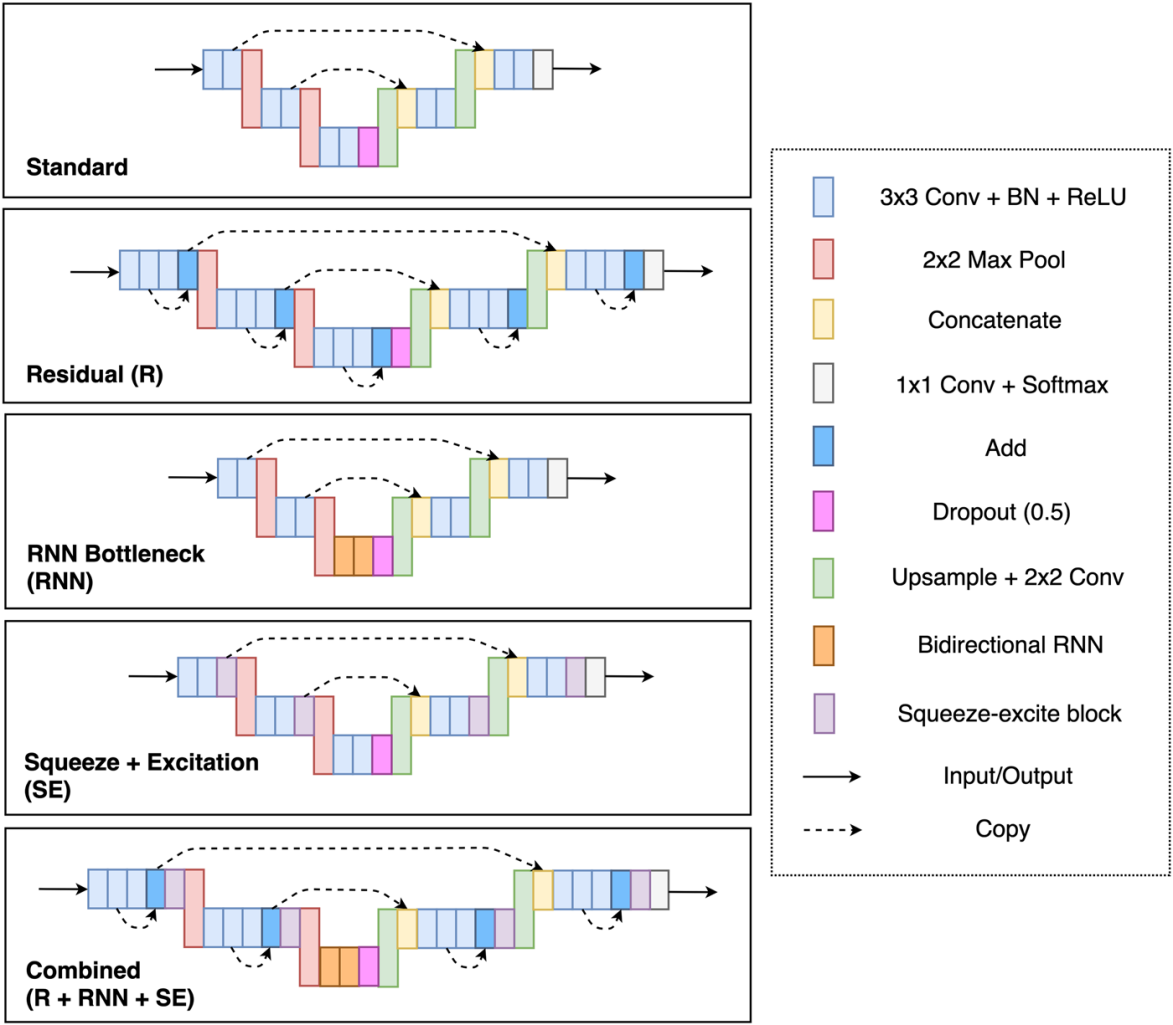
Illustration of the various network architectures used for the semantic segmentation method in this work. Due to space constraints, illustrated networks are shown with just two pooling layers, however this is by no means a restriction on the architectures. Note that the specific implementation of the squeeze-excite block may vary (one of cSE, sSE, scSE).

Semantic segmentation network architectures have evolved over time with a number of modifications proposed. Supplementary Table S3 summarises some of the key features presented, which are used to inform the choice of network architectures in this study. Building upon previous work^58,59^ in the area of semantic segmentation using fully-convolutional neural networks, the U-Net^60^ was proposed for biomedical image segmentation. Architectures based on the U-Net have been used previously for OCT retinal segmentation^22,23,31^, and as such, a similar standard U-net architecture (referred to as ‘Standard’) will be used in this work, along with a number of modified variants to assess the potential for performance improvement in choroidal segmentation. These modifications include the incorporation of residual learning^61–64^ (referred to as ‘Residual’), the replacement of the bottleneck with RNN layers^65^ (referred to as ‘RNN bottleneck’), and the addition of squeeze-excitation blocks^66–68^ (referred to as ‘Squeeze + Excitation’). Additionally, the combination of all three modifications is also considered (referred to as ‘Combined’). There are three squeeze and excitation block variants considered: spatial squeeze and channel excitation (cSE), channel squeeze and spatial excitation (sSE) and concurrent spatial and channel squeeze and channel excitation (scSE). Note that the ‘Combined’ network utilises the ‘scSE’ squeeze and excitation block variant. An illustration of each architecture used is provided in Fig. 2. Note that, in each network, convolutional layers incorporate zero-padding such that the input and output of each are the same size and no cropping is required. Batch-normalization^69^, is utilised at the input to each rectified linear unit in an effort to enhance training performance. A dropout of 50%^56^, is used at the output of the bottleneck of the network for regularisation. Each network used consists of four pooling layers and four up sampling layers. The first layer contains eight filters with this number doubled at each subsequent pooling layer and halved in a similar fashion for each up sampling layer.

#### Training

Each of the networks illustrated in Fig. 2 and described above are trained to perform semantic segmentation on full-size OCT images. To do this, a network is tasked with classifying each pixel in an image into one of four area classes. These area classes are defined as the vitreous (top of the image to ILM), retina (ILM to RPE), choroid (RPE to CSI) and sclera (CSI to bottom of the image). Therefore, each image has an associated area mask which is the target output for the FCNs. As described in set A in the data, 240 full-size OCT images are used for training while a separate 60 images are used for validation. For each column where at least one true boundary location is not present in the data (normally associated with shadows at the edge of some images), the corresponding column in the area mask is set to be the top area class (vitreous) and the same column in the image is zeroed. Due to the relatively small number of images, the data was augmented using horizontal flips (left to right/right to left). For each epoch, each image was randomly flipped horizontally with a 50% chance.

The Adam algorithm^57^, with default parameters $(\alpha =0.001,\,{\beta }_{1}=0.9,\,{\beta }_{2}=0.999,\,{\epsilon }=1\times {10}^{-8})$ is used for training to minimise the sum of cross-entropy loss and Dice overlap loss^70^. This loss combination is similar to that used in previous work^22^, although no additional weighting scheme is employed here. Each network is trained until convergence is observed with respect to the validation loss while the epoch with model with the highest validation accuracy (Dice overlap percentage) is chosen for evaluation. No early-stopping is employed, with convergence determined based on the inspection of the validation losses. No transfer learning is performed and no learning rate schedule is used. Instead, each network is trained from scratch with weights initialised with small random values.

### Image pre-processing

The choroid is a vascular layer of the eye. Its vascular nature, combined with the fact that is located behind a hyper-reflective layer (RPE), means that the contrast and visibility of the posterior boundary tends to be weak. The use of OCT image contrast enhancement techniques^71^, also known as attenuation coefficients^72^, was therefore considered in this work since it may improve the visibility of the boundaries, especially for the CSI, and also reduces the effect of shadows caused by the retinal blood vessels. This method has been used previously for improving visibility of the CSI^73^. The technique works under the assumption that local backscattering can be related to that of the corresponding attenuation, and therefore can be compensated. In this work the effect of the attenuation compensation was tested with two different network-input options; the standard OCT intensity image and the contrast enhanced (attenuation coefficient) equivalent.

### Boundary prediction and model evaluation

Given a scan and a trained network, probability maps for each of the boundaries can be calculated. For a patch-based method the probability maps are obtained by classifying patches centred on each pixel in the scan^20^. For a fully-convolutional method, the boundary probability maps are acquired by applying the Sobel filter to the area probability output of the FCN^37^. In both cases, the boundary positions may then be delineated by performing a graph search using Dijkstra’s shortest path algorithm^74^, where each pixel in the probability map corresponds to a vertex in the graph. This is inspired by the approach originally used by Chiu *et al*.^33^. Directed edges associated with each vertex are connected to neighbouring vertices to the immediate right (horizontally, diagonally above and diagonally below). To remove the need for manual start and end point initialisation, columns of maximum probability vertices, connected top to bottom, are appended to each end of the graph, with additional left to right connections made to the existing graph as required. The edge weights between each pair of vertices are determined by the respective probabilities and are given by Eq. (1).:

1 $$\begin{array}{c}{{\rm{w}}}_{{\rm{sd}}}=2-({{\rm{P}}}_{{\rm{s}}}+{{\rm{P}}}_{{\rm{d}}})+{{\rm{w}}}_{{\rm{\min }}}\end{array}$$

where P_s_ and P_d_ are the probabilities (0–1) of the source and destination vertices respectively, and ${{\rm{w}}}_{{\rm{\min }}}={1\times \mathrm{10}}^{-{\rm{5}}}$ is a small positive number added for system stability.

This step is performed using all scans in set B. To evaluate the performance, the delineated boundary positions for each image were compared to the true positions (the boundary position from manual segmentation of an expert human observer), from which the Dice overlap percentage is calculated for the four regions of interest, including the vitreous, retina, choroid, and sclera, as well as the mean pixel error and mean absolute pixel error (for the ILM, RPE and CSI) for each scan. Because the patch-based networks do not output area maps, Dice values cannot be calculated directly from the network output. Due to this and for the purposes of consistency between the methods, all Dice overlap values are calculated post-segmentation. Note that these values will be greater than Dice values obtained directly from the network output (in the semantic segmentation case) for cases where misclassifications do not affect the boundary errors.

In an effort to obtain a fair indication of the performance of the models, the full-width scans are used for input to the networks with a graph search performed on the corresponding full-size probability map. However, final error calculations and comparisons are only performed on a cropped region of all scans (approximately 100 pixels from the left and 250 pixels from the right) due to the presence of artefacts with this region (i.e. optic nerve head and shadows).

## Results

### Patch-based method results

**Figure 3:**
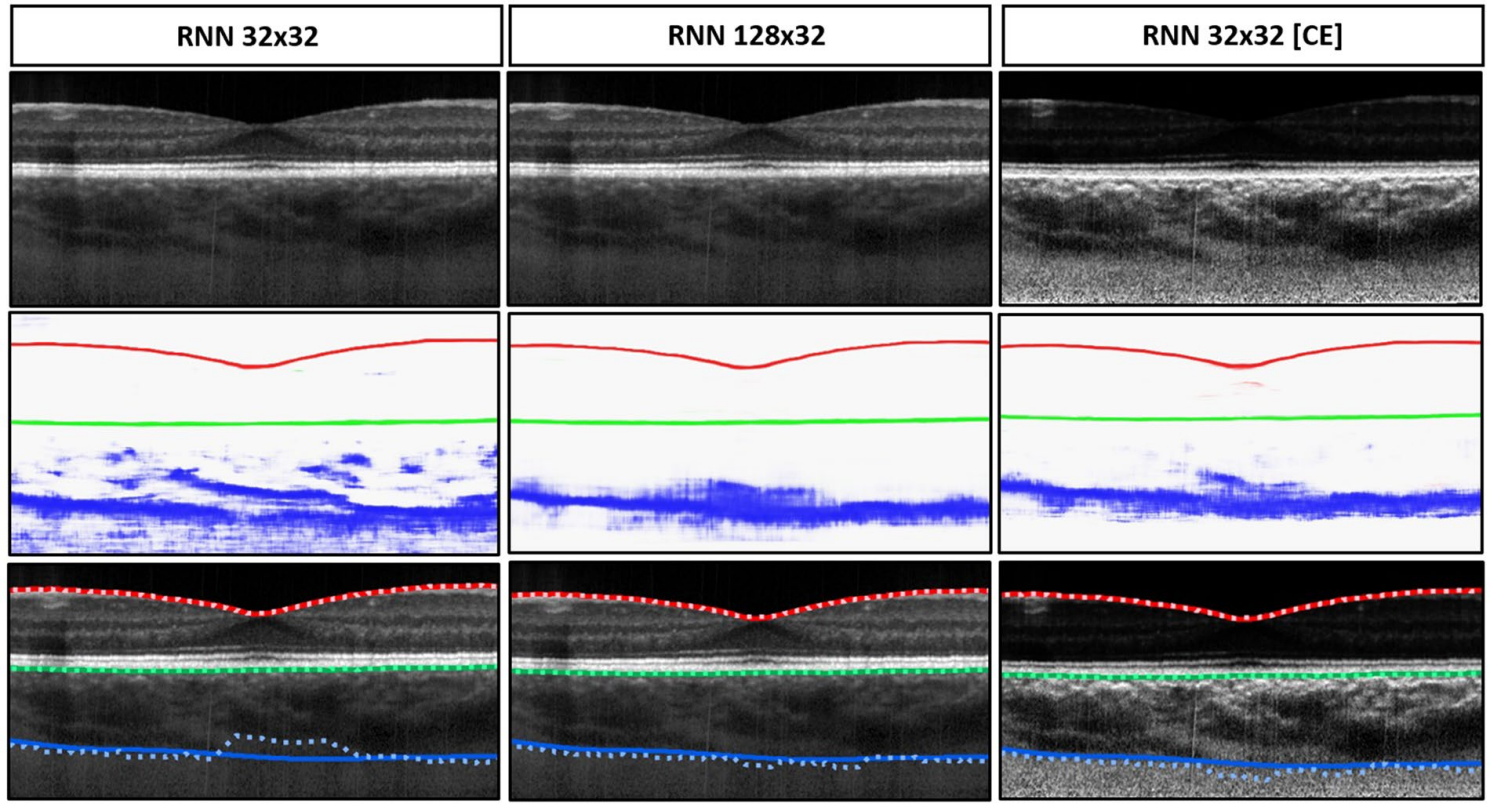
Example results for the segmentation of a single scan using the RNN 32 × 32, RNN 128 × 32, and RNN 32 × 32 with contrast enhancement (CE). From top to bottom: raw image, combined colour-coded probability maps of the three boundary classes, and boundary delineations where dotted lines are the predicted boundary locations and the solid lines indicate the true boundary locations. Each colour indicates a different boundary; red: ILM, green: RPE and blue: CSI. The effect of increasing the patch size and using contrast enhancement is evident with closer agreement between the true and predicted boundaries.

The Cifar CNN (CNN 1), Complex CNN (CNN 2) and RNN networks were trained using 32 × 32, 64 × 32, 64 × 64, and 128×32 patch sizes. All networks were additionally trained with contrast enhanced images for each patch size. The results for the dice overlap are summarised in Supplementary Table S4 and the boundary position errors in Table 1. For reference, evaluation is also performed with an automatic non-machine learning graph-search image-processing segmentation method, referred to below as automatic baseline^37^ on the same set of data (set B). Figure 3 illustrates results from a single example scan evaluated using an RNN. To assess the effects of the different architectures, patch size and the use of contrast enhancement on segmentation performance, a repeated measures ANOVA was also performed to examine the statistical significance of the differences in the mean absolute boundary errors associated with these factors. The networks converged in an average of 4.31 ± 5.54 epochs with a range of 2–20 epochs.

**Table 1:** Boundary position errors (in pixels) for each of the patch-based methods with comparison to the baseline. Mean error.

Method	ILM		RPE		CSI	
	ME	MAE	ME	MAE	ME	MAE
RNN (32 × 32)	−0.08 (0.25)	0.48 (0.10)	−0.19 (**0.18**)	**0.46** (**0.12**)	1.79 (4.84)	4.44 (4.23)
RNN (32 × 32) [CE]	**0.01** (0.24)	**0.45** (0.12)	−0.41 (**0.18**)	0.57 (0.13)	0.69 (3.06)	3.69 (2.27)
RNN (64 × 32)	−0.08 (0.24)	0.47 (0.12)	−0.35 (0.22)	0.54 (0.14)	0.35 (3.54)	3.81 (2.93)
RNN (64 × 32) [CE]	−0.22 (**0.23**)	0.52 (0.09)	−0.28 (0.22)	0.52 (0.13)	0.13 (2.57)	3.43 (1.97)
RNN (64 × 64)	−0.19 (0.24)	0.51 (0.09)	−0.43 (0.20)	0.59 (0.14)	0.42 (3.03)	3.36 (2.52)
RNN (64 × 64) [CE]	−0.16 (0.25)	0.50 (0.09)	−0.28 (0.22)	0.51 (0.15)	−**0.02** (2.71)	**3.23** (2.08)
RNN (128 × 32)	−0.08 (0.35)	0.50 (0.28)	−0.25 (0.22)	0.50 (0.13)	0.78 (3.31)	3.66 (2.85)
RNN (128 × 32) [CE]	−0.14 (0.30)	0.54 (0.19)	−0.19 (0.22)	0.49 (**0.12**)	0.07 (2.89)	3.48 (2.13)
CNN 1 (32 × 32)	−0.04 (0.31)	0.50 (0.25)	**0.03** (0.25)	0.50 (0.14)	2.17 (5.42)	5.05 (4.75)
CNN 1 (32 × 32) [CE]	−0.25 (0.25)	0.54 (**0.08**)	−0.31 (0.22)	0.53 (0.13)	1.10 (3.11)	3.73 (2.35)
CNN 1 (64 × 32)	−0.20 (0.25)	0.52 (0.09)	−0.30 (0.25)	0.54 (0.14)	1.18 (5.43)	4.56 (4.75)
CNN 1 (64 × 32) [CE]	−0.09 (0.25)	0.48 (0.10)	−0.40 (0.28)	0.61 (0.16)	−0.35 (2.68)	3.55 (1.95)
CNN 1 (64 × 64)	−0.09 (0.24)	0.49 (0.11)	0.14 (0.23)	0.48 (0.13)	1.17 (4.01)	3.67 (3.51)
CNN 1 (64 × 64) [CE]	−0.14 (0.24)	0.50 (0.10)	−0.35 (0.30)	0.58 (0.17)	0.01 (3.29)	3.35 (2.79)
CNN 1 (128 × 32)	−0.31 (0.25)	0.57 (0.09)	0.08 (0.30)	0.51 (0.13)	0.43 (3.07)	3.61 (2.61)
CNN 1 (128 × 32) [CE]	−0.04 (0.24)	0.47 (0.10)	−0.22 (0.33)	0.57 (0.16)	1.04 (**2.45**)	3.43 (**1.89**)
CNN 2 (32 × 32)	**0.01** (0.55)	0.51 (0.51)	−0.44 (**0.18**)	0.59 (0.13)	1.26 (4.69)	4.72 (3.95)
CNN 2 (32 × 32) [CE]	−0.14 (**0.23**)	0.49 (0.09)	−0.23 (0.22)	0.51 (0.13)	0.20 (3.35)	3.78 (2.39)
CNN 2 (64 × 32)	−0.13 (0.24)	0.50 (0.09)	−0.40 (0.20)	0.57 (0.14)	1.32 (4.24)	4.25 (3.58)
CNN 2 (64 × 32) [CE]	−0.26 (**0.23**)	0.53 (0.09)	−0.65 (0.25)	0.77 (0.18)	0.25 (2.86)	3.57 (2.14)
CNN 2 (64 × 64)	−0.30 (**0.23**)	0.55 (0.10)	−0.39 (0.20)	0.57 (0.14)	0.77 (4.60)	4.12 (3.97)
CNN 2 (64 × 64) [CE]	−0.19 (0.25)	0.54 (0.09)	−0.53 (0.27)	0.68 (0.18)	0.54 (3.36)	3.54 (2.69)
CNN 2 (128 × 32)	−0.15 (**0.23**)	0.49 (0.10)	−0.40 (0.19)	0.57 (0.13)	1.78 (4.10)	4.18 (3.38)
CNN 2 (128 × 32) [CE]	−0.19 (0.29)	0.56 (0.18)	−0.31 (0.24)	0.58 (0.13)	0.86 (2.98)	3.61 (2.34)
Baseline^37^	−0.27 (0.41)	0.58 (0.36)	−1.14 (0.65)	1.23 (0.60)	−3.64 (8.62)	5.82 (7.77)

(ME) and mean absolute error (MAE) are reported in terms of mean value and (per B-scan standard deviation) for each of the three boundaries. [CE] indicates that the network was trained and tested with images pre-processed using contrast enhancement. CNN 1: Cifar CNN, CNN 2: Complex CNN. The best result for each boundary is highlighted in bold text.

All patch-based methods perform comparably on the vitreous with mean dice overlaps of approximately 99.80% and standard deviations between 0.05 and 0.20 (Supplementary Table S4). For the retina, the dice overlaps of all machine learning methods were again comparable and ranged between 99.19% and 99.41% with standard deviations between 0.10 and 0.20. Overall, the machine learning methods performed noticeably better than the automatic baseline on the retina. The results for the sclera and retina translate directly to the similarities observable for the ILM and RPE boundary position errors with similar mean absolute errors for all methods of approximately 0.50 pixels for the ILM and between 0.46 and 0.77 pixels for the RPE.

Although the difference in performance of the methods on the ILM and RPE boundaries is marginal, there were statistically significant differences between some of the methods. The RNN yielded significantly smaller mean absolute errors (p < 0.01) compared to the other two architectures for both the ILM and RPE boundaries. In addition, a lack of contrast enhancement provided significantly lower error (p < 0.01) for the RPE, while there was no significant effect of contrast enhancement for the segmentation performance for the ILM. In terms of patch size, for the ILM boundary, 32 × 32 patches yielded significantly lower error (p < 0.01) than 128 × 32 patches but were not significantly different to the 64 × 32 or 64 × 64 variants. For the RPE boundary, 32 × 32 and 128 × 32 patches both showed significantly lower error (p < 0.01) than 64 × 32 and 64 × 64 patches, however there was no significant difference between 32 × 32 and 128 × 32 patches (p > 0.05).

The dice overlaps for both the choroid and sclera as well as the boundary position error for the CSI showed greater variability between the various methods. Here, the architecture, patch size and effect of contrast enhancement all exhibited statistically significant effects on performance. Overall, the RNN architecture exhibited the lowest error on the CSI boundary with an average of 3.64 pixels (average of the eight methods) mean absolute error compared to 3.74 and 3.97 pixels for the Cifar and Complex CNN respectively, which was statistically significant (p < 0.01). Using contrast enhanced images also yielded significantly lower CSI boundary mean absolute error overall with an average of 3.53 pixels compared to 4.12 pixels without (difference of 0.59 pixels) (p < 0.01). Of the patch sizes, the 64 × 64 showed the lowest error with an average CSI mean absolute error of 3.55 pixels. This was significantly lower (p < 0.01) than the 32 × 32 (4.24 pixels) and 64 × 32 (3.86 pixels) patch sizes but not significantly different to the 128 × 32 (3.66 pixels).

**Figure 4:**
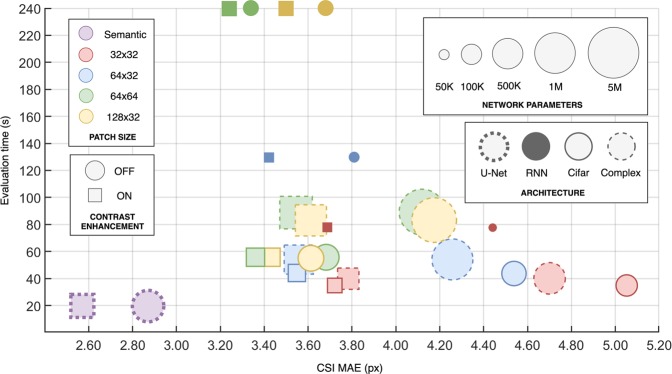
Accuracy vs. Speed vs. Complexity comparison of the patch-based methods and a semantic segmentation method using the standard U-net architecture. The different network architectures and patch sizes are compared as is the use of contrast enhancement. Here, the accuracy (CSI mean absolute error in pixels [x-axis]) is compared to the network complexity (number of parameters [shape size]) and the evaluation time (per B-scan in seconds [y-axis]). Due to high similarity in accuracy, speed and parameters, only a single semantic segmentation architecture (standard U-net) is illustrated here.

For a complete comparison of all the patch-based methods, the per B-scan evaluation time (speed) and number of network parameters (complexity) is reported against the CSI boundary mean absolute error for each architecture. A complete visual comparison of each method’s performance is provided in Fig. 4. It is evident that the RNN architecture is the simplest (fewest parameters) but also the slowest (longest per B-scan evaluation time) while the Cifar CNN was the fastest and the Complex CNN possessed the most parameters.

### Semantic segmentation method results

**Figure 5:**
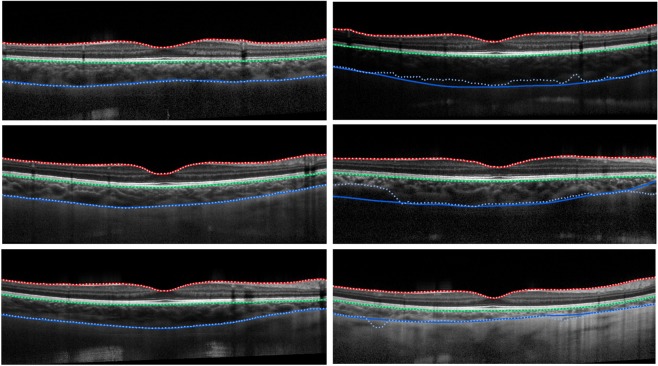
Example segmentations for the standard U-Net architecture (no contrast enhancement). Solid lines correspond to the true boundary positions and the dotted lines represent the predicted locations. Left: three cases of successful delineation of all boundaries with close agreement between the predictions and truths. Right: three cases of failure on the CSI boundary with observable differences between the prediction and truth.

Each of the semantic segmentation networks depicted in Fig. 2 were trained and evaluated as described in the Methods section. Like the patch-based methods, all networks were trained and evaluated using contrast-enhanced images in addition to the raw images. Results for the dice overlap are presented in Supplementary Table S5 while the boundary position errors are reported in Table 2. Using the mean absolute boundary errors, a repeated measures ANOVA was performed to examine the statistical significance of any differences in performance between the methods. Figure 5 presents some example segmentations using the standard U-net architecture (without contrast enhancement). The networks converged in an average of 77.57 ± 18.46 epochs with a range of 34–98 epochs.

**Table 2:** Boundary position errors (in pixels) for each of the semantic segmentation methods with comparison to the baseline.

Method	ILM		RPE		CSI	
	ME	MAE	ME	MAE	ME	MAE
Standard	−0.07 (**0.22**)	0.51 (0.10)	−0.10 (0.19)	0.45 (0.12)	0.85 (2.45)	2.86 (2.01)
Standard [CE]	0.07 (0.25)	0.51 (0.11)	−**0.03** (0.20)	0.45 (0.11)	0.19 (2.24)	2.58 (1.65)
Residuals	0.05 (0.23)	**0.50** (0.11)	−0.14 (0.19)	0.45 (0.11)	**0.02** (2.31)	2.59 (1.58)
Residuals [CE]	−0.21 (0.24)	0.53 (0.09)	−0.09 (0.29)	0.46 (0.22)	0.68 (**2.05**)	**2.53** (1.52)
RNN	−0.25 (**0.22**)	0.55 (0.09)	−0.27 (0.19)	0.49 (0.13)	1.05 (2.35)	2.56 (1.89)
RNN [CE]	−0.11 (0.24)	0.51 (0.09)	−0.25 (0.19)	0.48 (0.12)	0.42 (2.42)	2.59 (1.92)
cSE	**0.02** (0.68)	0.54 (0.63)	−0.08 (**0.18**)	**0.44** (0.11)	0.59 (2.53)	2.73 (1.97)
cSE [CE]	−0.15 (0.28)	0.52 (0.16)	−0.21 (0.21)	0.47 (0.12)	0.41 (2.10)	2.57 (1.57)
sSE	−0.03 (**0.22**)	0.51 (**0.08**)	−0.16 (0.18)	0.46 (0.12)	**0.02** (2.61)	2.84 (1.83)
sSE [CE]	−0.08 (0.36)	0.52 (0.26)	−0.27 (0.21)	0.50 (0.13)	0.81 (2.33)	2.72 (1.84)
scSE	−0.04 (0.33)	0.53 (0.24)	−0.16 (0.20)	0.46 (0.12)	0.34 (2.30)	2.60 (1.68)
scSE [CE]	−0.16 (0.23)	0.52 (0.09)	0.13 (0.20)	0.46 (0.11)	0.19 (2.20)	2.60 (**1.51**)
Combined	0.06 (**0.22**)	**0.50** (0.09)	−0.07 (0.20)	**0.44** (0.12)	1.10 (2.71)	2.69 (2.14)
Combined [CE]	−0.03 (0.24)	0.51 (0.09)	−0.19 (0.20)	0.47 (0.12)	1.25 (2.06)	**2.53** (1.52)
Baseline^37^	−0.27 (0.41)	0.58 (0.36)	−1.14 (0.65)	1.23 (0.60)	−3.64 (8.62)	5.82 (7.77)

Mean error (ME) and mean absolute error (MAE) are reported in terms of mean value and (per B-scan standard deviation) for each of the three boundaries. [CE] indicates that the network was trained and tested with images pre-processed using contrast enhancement. The best result for each boundary is highlighted in bold text.

The results for the dice overlap are similar across all semantic segmentation methods for all regions. The difference between the best and worst performing methods was small with just 0.02% difference for the vitreous, 0.06% for the retina, 0.18% for the choroid, and 0.09% for the sclera. A similar trend is observable for the mean absolute boundary position errors with a difference of just 0.05 pixels mean absolute error observed between the best and worst performing methods on the ILM and RPE boundaries. There was slightly more variability in the results for the CSI with a range of 0.33 pixels mean absolute error. Notably, all machine learning methods performed substantially better than the automatic baseline on the RPE and CSI with respect to both accuracy and consistency with a relatively smaller improvement observed on the ILM.

Overall, there were no statistically significant effects of architecture or contrast enhancement for the mean absolute errors of the ILM and CSI boundaries. For the RPE boundary, the standard architecture yielded the lowest average mean absolute error which was significantly lower (p < 0.01) than that of the RNN bottleneck, sSE and scSE architectures. However, the difference in errors was small for each of these (<0.05 pixels). Contrast enhancement also had a significant effect (p < 0.001) with smaller mean absolute boundary errors for the RPE but the improvement was small (<0.02 pixels).

## Discussion

**Figure 6:**
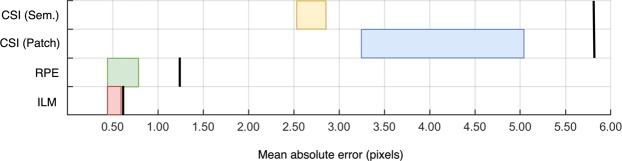
Accuracy comparison for the three boundaries of interest. The range of mean absolute errors for all machine learning methods is shown for each boundary (range indicated by each coloured box). RPE and ILM boxes contain both sematic and patch-based methods, while CSI has two separate boxes for each of the methods. Each boundary is compared to the automatic baseline method indicated by a solid black line along the same row.

This paper has examined a number of supervised deep learning methods for the task of retinal and choroidal segmentation in OCT images. Here, both patch-based methods and semantic segmentation methods were considered with each compared to an automatic baseline method. The effect of patch size (for the patch-based methods), network architecture and contrast enhancement were analysed. The deep learning methods gave superior performance on all boundaries compared to a standard image analysis method used as a baseline. Overall, the findings suggest that all machine learning methods exhibit similar accuracy and good performance on the retinal layers (ILM and RPE) while performance on the CSI showed more variability between methods. This is likely linked to the well-defined ILM and RPE boundaries in comparison with the CSI. This relative performance between the boundaries is illustrated in Fig. 6.

For the patch-based methods; changes in architecture and patch size as well as the use of contrast enhancement had a significant effect on the CSI boundary error. Contrast enhancement reduced the CSI mean absolute error as a result of the additional emphasis applied to the boundary. The performance benefit of increasing the patch size can be attributed to the additional context available around each pixel, allowing the networks to more easily classify each individual patch. In terms of architecture, the RNNs exhibited lower CSI errors than the corresponding CNNs, in general. Despite possessing the fewest parameters, the RNNs were considerably slower than the CNNs due to the large number of operations required to pass over the images pixels sequentially.

For the semantic segmentation methods; the change in architecture and the use of contrast enhancement had less noticeable effects on the CSI with just 0.33 pixels mean absolute error separating the best and worst performing methods. In contrast, the corresponding range of the patch-based methods was 1.82 pixels. Overall, the semantic segmentation methods performed comparably to one another in terms of accuracy, evaluation speed as well as complexity. However, compared to the patch-based methods, they performed noticeably better on the CSI boundary with a mean absolute error for the best performing method of 2.53 pixels compared to 3.23 pixels. This improvement can be attributed to the additional context available to the network as the whole image is processed at once. The semantic segmentation methods were also considerably faster, taking approximately 20 seconds per B-scan as opposed to approximately 35–240 seconds for the various patch-based methods. Figure 4 illustrates a comparison between the patch-based methods and the semantic segmentation method (using the standard U-net architecture). The comparison shows that for OCT image segmentation, patch-based methods are not of significant benefit given the slower evaluation and higher error.

It is worth noting that the different architectural changes introduced for the semantic segmentation did not show a significant effect on the performance. This is possibly due to the lack of overall depth (number of layers) of the network architecture. In particular, residual networks were introduced to improve the performance of very deep networks and potentially have minimal impact otherwise. Additionally, it is possible that the performance here is not limited by the architecture. For example, the performance may be constrained by the richness of the data, the loss function and/or the optimizer used among other aspects.

There exists a vast number of possible combinations of parameters (architectural and otherwise) that can be tested, far too many than are feasible to include in this work. Future work may extend the findings here and investigate other changes in the methodology. For instance, activation functions such as Leaky ReLU^75,76^, Parametric ReLU^75,77^, Randomized Leaky ReLU^75^, and Flexible ReLU^78^ have been proposed as improvements to the standard ReLU and may be considered. Loss functions such as Tverksy loss^79^ may be used to address data imbalance while a loss function may be designed or modified with the goal to better discriminate boundary transitions^22^. Given the promising performance of Adam, variants including Nadam^80^ and Adamax^57^ may be useful alternatives for training while additional performance may be gained from optimally tuning the dropout values^56^. Other parameters such as kernel size, number of convolutional layers and number of pooling layers may also be considered. For instance, ReLayNet^22^ utilised a single 7 × 3 convolutional block for each of three pooling layers while Venhuizen *et al*.^23^ utilised two 3 × 3 blocks for each of six pooling layers.

Given the low error and high consistency on retinal boundaries such as the RPE and ILM, future work in the area should focus on the more challenging CSI boundary. In particular, methods utilising semantic segmentation seem promising and appear to provide superior accuracy and speed to a patch-based approach. For volumetric data, this idea can be extended by including adjacent B-scans to introduce additional context^81^. There is also potential benefit in improving or even replacing the graph search component of these methods. Ideally, an end-to-end ML approach could be adopted which outputs per-boundary positions or, to ensure correct layer topology, the thicknesses of each layer^82^. Another option to consider is transfer learning^83,84^, using pre trained weights, which may help to improve performance particularly in the case of insufficient data. Additional augmentations (e.g. rotations, noise, contrast) may also be used to build a richer training set. The findings presented here may be used to inform future work in the area of chorio-retinal boundary analysis in OCT images. Future studies should explore how these methods will perform in other OCT modalities, particularly swept-source OCT that has demonstrated a superior performance to visualize the deeper choroidal layer^85^ compared to spectral domain OCT used in this study. It is worth noting that the images used in the current study are from young healthy participants, and therefore further work is required to examine these segmentation methods in cases of ocular pathology and in older populations.

Since most of the commercially available OCT instruments do not provide methods for automatic choroidal segmentation and the use of deep learning methods for choroidal segmentation is still largely unexplored, this work demonstrates the potential of these techniques and the advantage (superior performance) over standard image analysis methods. Thus, the methods presented here are likely to have a positive impact on clinical and research tasks involving OCT choroidal segmentation.

## Supplementary information

SUPPLEMENTARY INFO

## Data Availability

The datasets analysed during the current study are currently not publicly available. However, the algorithms developed in this work are available from the corresponding author on reasonable request.

## References

[1] Nickla DL, Wallman J (2010). The multifunctional choroid. Prog. Retin. Eye. Res..

[2] Mrejen S, Spaide RF (2013). Optical coherence tomography: imaging of the choroid and beyond. Surv. Ophthalmol..

[3] Huang D (1991). Optical coherence tomography. Science.

[4] Spaide RF, Koizumi H, Pozzoni MC (2008). Enhanced depth imaging spectral-domain optical coherence tomography. Am. J. Ophthalmol..

[5] Read SA, Collins MJ, Vincent SJ, Alonso-Caneiro D (2013). Choroidal thickness in childhood. Invest. Ophthalmol. Vis. Sci..

[6] Read SA, Collins MJ, Vincent SJ, Alonso-Caneiro D (2015). Macular retinal layer thickness in childhood. Retina.

[7] Grover S, Murthy RK, Brar VS, Chalam KV (2009). Normative data for macular thickness by high-definition spectral-domain optical coherence tomography (Spectralis). Am. J. Ophthalmol..

[8] Margolis R, Spaide RF (2009). A pilot study of enhanced depth imaging optical coherence tomography of the choroid in normal eyes. Am. J. Ophthalmol..

[9] Harb E, Hyman L, Fazzari M, Gwiazda J, Marsh-Tootle W (2012). Factors associated with macular thickness in the COMET myopic cohort. Optom. Vis. Sci..

[10] Read SA, Collins MJ, Vincent SJ, Alonso-Caneiro D (2013). Choroidal thickness in myopic and nonmyopic children assessed with enhanced depth imaging optical coherence tomography. Invest. Ophthalmol. Vis. Sci..

[11] Sakamoto A (2010). Three-dimensional imaging of the macular retinal nerve fiber layer in glaucoma with spectral-domain optical coherence tomography. Invest. Ophthalmol. Vis. Sci..

[12] Wood A (2011). Retinal and choroidal thickness in early age-related macular degeneration. Am. J. Ophthalmol..

[13] Bussel II, Wollstein G, Schuman JS (2014). OCT for glaucoma diagnosis, screening and detection of glaucoma progression. Br. J. Ophthalmol..

[14] Medina FJL (2012). Use of nonmydriatic spectral-domain optical coherence tomography for diagnosing diabetic macular edema. Am. J. Ophthalmol..

[15] Fung AE (2007). An optical coherence tomography-guided, variable dosing regimen with intravitreal ranibizumab (Lucentis) for neovascular age-related macular degeneration. Am. J. Ophthalmol..

[16] Baghaie A, Yu Z, D’Souza RM (2015). State-of-the-art in retinal optical coherence tomography analysis. Quant. Imaging Med. Surg..

[17] DeBuc, D. C. A review of algorithms for segmentation of retinal image data using optical coherence tomography in *Image Segmentation* (ed. Ho, P. G.) 15–54 (InTech, 2011).

[18] Vermeer K, V der Schoot J, Lemij H, De Boer J (2011). Automated segmentation by pixel classification of retinal layers in ophthalmic OCT images. Biomed. Opt. Express.

[19] Srinivasan PP, Heflin SJ, Izatt JA, Arshavsky VY, Farsiu S (2014). Automatic segmentation of up to ten layer boundaries in SD-OCT images of the mouse retina with and without missing layers due to pathology. Biomed. Opt. Express.

[20] Fang L (2017). Automatic segmentation of nine retinal layer boundaries in OCT images of non-exudative AMD patients using deep learning and graph search. Biomed. Opt. Express.

[21] Lang A (2013). Retinal layer segmentation of macular OCT images using boundary classification. Biomed. Opt. Express.

[22] Roy AG (2017). ReLayNet: Retinal layer and fluid segmentation of macular optical coherence tomography using fully convolutional network. Biomed. Opt. Express.

[23] Venhuizen FG (2017). Robust total retina thickness segmentation in optical coherence tomography images using convolutional neural networks. Biomed. Opt. Express.

[24] Devalla SK (2018). DRUNET: a dilated-residual U-Net deep learning network to segment optic nerve head tissues in optical coherence tomography images. Biomed. Opt. Express.

[25] Shah, A., Abramoff, M. & Wu, X. Simultaneous multiple surface segmentation using deep learning in *Deep Learning in Medical Image Analysis and Multimodal Learning for Clinical Decision Support* (ed. Cardoso, J. *et al*.) 3–11 (Springer, 2017).

[26] Xu Y (2017). Dual-stage deep learning framework for pigment epithelium detachment segmentation in polypoidal choroidal vasculopathy. Biomed. Opt. Express.

[27] Loo J, Fang L, Cunefare D, Jaffe GJ, Farsiu S (2018). Deep longitudinal transfer learning-based automatic segmentation of photoreceptor ellipsoid zone defects on optical coherence tomography images of macular telangiectasia type 2. Biomed. Opt. Express.

[28] McDonough, K., Kolmanovsky, I. & Glybina I. V. A neural network approach to retinal layer boundary identification from optical coherence tomography images in *Proceedings of 2015 IEEE conference on Computational Intelligence in Bioinformatics and Computational Biology* 1–8 (IEEE, 2015).

[29] Cicek, O., Abdulkadir, A., Lienkamp, S. S., Brox, T. & Ronneberger, O. 3d U-Net: Learning Dense Volumetric Segmentation from Sparse Annotation in *Proceedings of the International Conference on Medical Image Computing and Computer-Assisted Intervention –* MICCAI 2016 (ed. Ourselin, S., Joskowicz, L., Sabuncu, M. R., Unal, G. & Wells, W.) 424–432 (Springer, 2016).

[30] Hamwood J, Alonso-Caneiro D, Read SA, Vincent SJ, Collins MJ (2018). Effect of patch size and network architecture on a convolutional neural network approach for automatic segmentation of OCT retinal layers. Biomed. Opt. Express.

[31] Kugelman J, Alonso-Caneiro D, Read SA, Vincent SJ, Collins MJ (2018). Automatic segmentation of OCT retinal boundaries using recurrent neural networks and graph search. Biomed. Opt. Express.

[32] Alonso-Caneiro, D. *et al*. Automatic retinal and choroidal boundary segmentation in OCT images using patch-based supervised machine learning methods in *Computer Vision – ACCV 2018 Workshops*. (ed. Carneiro, G. & You. S) 215–228 (Springer, Cham, 2019).

[33] Chiu SJ (2010). Automatic segmentation of seven retinal layers in SDOCT images congruent with expert manual segmentation. Opt. Express.

[34] Kajic V (2012). Automated choroidal segmentation of 1060 nm OCT in healthy and pathologic eyes using a statistical model. Biomed. Opt. Express.

[35] Zhang L (2012). Automated segmentation of the choroid from clinical SD-OCT. Invest. Ophthalmol. Vis. Sci..

[36] Tian J, Marziliano P, Baskaran M, Tun TA, Aung T (2013). Automatic segmentation of the choroid in enhanced depth imaging optical coherence tomography images. Biomed. Opt. Express.

[37] Alonso-Caneiro D, Read SA, Collins MJ (2013). Automatic segmentation of choroidal thickness in optical coherence tomography. Biomed. Opt. Express.

[38] Hussain MA (2018). An automated method for choroidal thickness measurement from Enhanced Depth Imaging Optical Coherence Tomography images. Comput. Med. Imaging Graph..

[39] Twa MD, Schulle KL, Chiu SJ, Farsiu S, Berntsen DA (2016). Validation of macular choroidal thickness measurements from automated SD-OCT image segmentation. Optom. Vis. Sci..

[40] Uppugunduri Sushmitha Rao, Rasheed Mohammed Abdul, Richhariya Ashutosh, Jana Soumya, Chhablani Jay, Vupparaboina Kiran Kumar (2018). Automated quantification of Haller’s layer in choroid using swept-source optical coherence tomography. PLOS ONE.

[41] Philip AM (2016). Choroidal thickness maps from spectral domain and swept source optical coherence tomography: algorithmic versus ground truth annotation. Br. J. Ophthalmol..

[42] Sui X (2017). Choroid segmentation from optical coherence tomography with graph-edge weights learned from deep convolutional neural networks. Neurocomputing.

[43] Chen, M., Wang, J., Oguz, I., VanderBeek, B. L. & Gee, J. C. Automated segmentation of the choroid in EDI-OCT images with retinal pathology using convolution neural networks in *Fetal, Infant and Ophthalmic Med. Image Anal*. (ed. Cardoso, J. *et al*.) 177–184 (Springer, 2017). 10.1007/978-3-319-67561-9_20 PMC5947958 29757338

[44] Badrinarayanan, V., Kendall, A. & Cipolla, R. SegNet: A Deep Convolutional encoder-decoder architecture for image segmentation. *CoRR***abs/1511.00561**, https://arxiv.org/abs/1511.00561 (2015). 10.1109/TPAMI.2016.2644615 28060704

[45] Al-Bander, B., Williams, B. M., Al-Taee, M. A., Al-Nuaimy, W. & Zheng, Y. A novel choroid segmentation method for retinal diagnosis using deep learning in *2017 10th International Conference on Developments in eSystems Engineering* (DeSE) (ed. Hamdan, H., Al-Jumeily, D., Hussain, A., Tawfik, H. & Hind, J.) 182–187 (IEEE, 2017).

[46] Chollet, F. *Keras*, https://github.com/fchollet/keras (2015).

[47] Abadi, M. *et al*. Tensorflow: Large-scale machine learning on heterogeneous systems, https://tensorflow.org (2015).

[48] Rawat W, Wang Z (2017). Deep Convolutional Neural Networks for Image Classification: A Comprehensive Review. Neural Comput..

[49] Garcia-Garcia, A., Orts-Escolano, S., Oprea, S., Villena-Martinez, V. & Garcia-Rodriguez, J. A Review on Deep Learning Techniques Applied to Semantic Segmentation. *CoRR***abs/1704.06857**, https://arxiv.org/abs/1704.06857 (2017).

[50] Nair, V. & Hinton, G. E. Rectified Linear Units Improve Restricted Boltzmann Machines in *Proceedings of the 27th International Conference on Machine Learning* (ICML, 2010).

[51] Glorot, X., Bordes, A. & Bengio. Y. Deep sparse rectifier neural networks in *Proceedings of the 14th International Conference on Artificial Intelligence and Statistics*. 315–323 (PMLR, 2011).

[52] Graves, A. & Jaitly, N. Towards end-to-end speech recognition with recurrent neural networks in *Proceedings of the 31st International Conference on Machine Learning – Volume 32*, 1764–1772 (JMLR.org, 2014).

[53] Graves, A., Mohamed, A. & Hinton G. Speech recognition with deep recurrent neural networks in *Proceedings of 2013 IEEE International Conference on Acoustics, Speech and Signal Processing*. 6645–6649 (IEEE, 2013).

[54] Graves A (2009). A Novel Connectionist system for Unconstrained Handwriting Recognition. IEEE Trans. Pattern Anal. Mach. Intell..

[55] Visin, F. *et al*. Renet: A recurrent neural network based alternative to convolutional networks. *CoRR***abs/1505.00393**, https://arxiv.org/abs/1505.00393 (2015).

[56] Srivastava N, Hinton G, Krizhevsky A, Sutskever I, Salakhutdinov R (2014). Dropout: a simple way to prevent neural networks from overfitting. J. Mach. Learn. Res..

[57] Kingma, D. P. & Ba, J. Adam: A method for stochastic optimization. *CoRR***abs/1412.6980**, https://arxiv.org/abs/1505.00393 (2014).

[58] Shelhamer E, Long J, Darrell T (2016). Fully Convolutional networks for semantic segmentation. IEEE Trans. Pattern Anal. Mach. Intell..

[59] Noh, H., Hong, S. & Han, B. Learning deconvolution network for semantic segmentation in *Proceedings of the 2015 IEEE International Conference on Computer Vision*. 1520-1528 (IEEE Computer Society, 2015).

[60] Ronneberger, O., Fischer, P. & Brox, T. U-Net: Convolutional Networks for Biomedical Image Segmentation. *CoRR***abs/1505.04597**, https://arxiv.org/abs/1505.04597 (2015).

[61] He, K., Zhang, X., Ren, S. & Sun, J. Deep residual learning for image recognition in *2016 IEEE Conference on Computer Vision and Pattern Recognition (IEEE, 2016)*.

[62] He, K., Zhang, X., Ren, S. & Sun, J. Identity mappings in deep residual networks in *2016 European Conference on Computer Vision*. 630–645 (Springer, 2016).

[63] Drozdzal, M., Vorontsov, E., Chartrand, G., Kadoury, S. & Pal, C. The importance of skip connections in biomedical image segmentation. *CoRR***abs/1608.04117**, https://arxiv.org/abs/1608.04117 (2016).

[64] Zhang Z, Liu Q, Wang Y (2018). Road extraction by deep residual U-Net. IEEE Geosci. and Remote Sens. Lett..

[65] Visin, F. *et al*. ReSeg: A recurrent neural network-based model for semantic segmentation. *CoRR***abs/1511.07053**, https://arxiv.org/abs/1511.07053 (2016).

[66] Hu, J., Shen, L. & Sun, G. Squeeze-and-excitation networks. *CoRR***abs/1709.01507**, https://arxiv.org/abs/1709.01507 (2017).

[67] Roy, A.G., Navab, N. & Wachinger, C. Concurrent Spatial and Channel Squeeze & Excitation in Fully Convolutional Networks. *CoRR***abs/1803.02579**, https://arxiv.org/abs/1803.02579 (2018). 10.1109/TMI.2018.2867261 30716024

[68] Roy, A. G., Navab, N. & Wachinger, C. Recalibrating fully convolutional networks with spatial and channel ‘squeeze & excitation’ blocks. *CoRR****abs/1808.08127***, https://arxiv.org/abs/1808.08127 (2018). 10.1109/TMI.2018.2867261 30716024

[69] Ioffe, S. & Szegedy, C. Batch Normalization: Accelerating deep network training by reducing internal covariate shift. *CoRR***abs/1502.03167**, https://arxiv.org/abs/1502.03167 (2015).

[70] Sudre, C. H., Li, W., Vercauteren, T., Ourselin, S. & Jorge Cardoso, M. Generalised dice overlap as a deep learning loss function for highly unbalanced segmentations. *CoRR***abs/1707.03237**, https://arxiv.org/abs/1707.03237 (2017). 10.1007/978-3-319-67558-9_28 PMC7610921 34104926

[71] Girard MJ, Strouthidis NG, Ethier CR, Mari JM (2011). Shadow removal and contrast enhancement in optical coherence tomography images of the human optic nerve head. Invest. Ophthalmol. Vis. Sci..

[72] Vermeer K, Mo J, Weda J, Lemij H, De Boer J (2014). Depth-resolved model based reconstruction of attenuation coefficients in optical coherence tomography. Biomed. Opt. Express.

[73] Gupta P (2014). A simplified method to measure choroidal thickness using adaptive compensation in enhanced depth imaging optical coherence tomography. PLoS ONE.

[74] Dijkstra EW (1959). A note on two problems in connexion with graphs. Numer. Math..

[75] Xu, B. *et al*. Empirical evaluation of rectified activations in convolutional network. *CoRR***abs/1505.00853**, https://arxiv.org/abs/1505.00853 (2015).

[76] Maas, A. L., Hannun, A. Y. & Ng, A. Y. Rectifier nonlinearities improve neural network acoustic models in *Proceedings of the International Conference on Machine Learning* (ICML, 2013).

[77] He, K., Zhang, X., Ren, S. & Sun, J. Delving deep into rectifiers: surpassing human-level performance on imagenet classification. *CoRR***abs/1502.01852**, https://arxiv.org/abs/1502.01852 (2015).

[78] Qiu, S., Xu, X. & Cai, B. FReLU: Flexible Rectified Linear Units for Improving Convolutional Neural Networks. *CoRR***abs/1706.08098**, https://arxiv.org/abs/1706.08098 (2017).

[79] Salehi, S. S. M., Erdogmus D. & Gholipour, A. Tversky loss function for image segmentation using 3D fully convolutional deep networks. *CoRR***abs/1706.05721**, https://arxiv.org/abs/1706.05721 (2017).

[80] Dozat, T. Incorporating Nesterov Momentum into Adam in *Proceedings of the International Conference on Learning Representation* (ICLR, 2016).

[81] Milletari, F., Navab, N. & Ahmadi, S. V-Net: Fully convolutional neural networks for volumetric medical image segmentation. *CoRR***abs/1606.04797**, https://arxiv.org/abs/1606.04797 (2016).

[82] He, Y. *et al*. Topology guaranteed segmentation of the human retina from OCT using convolutional neural networks. *CoRR***abs/1803.05120**, https://arxiv.org/abs/1803.05120 (2018).

[83] Yosinski, J., Clune, J. & Bengjo, Y. How transferable are features in deep neural networks? in *Proceedings of the 27th International Conference on Neural Information Processing Systems - Volume 2*, 3320–3328 (MIT Press, 2014).

[84] Tan, C. *et al*. A survey on deep transfer learning. *CoRR***abs/1808.01974**, https://arxiv.org/abs/1808.01974 (2018).

[85] Chandrasekera E, Wong EN, Sampson DM, Alonso-Caneiro D, Chen FK (2018). Posterior choroidal stroma reduces accuracy of automated segmentation of outer choroidal boundary in swept source optical coherence tomography. Invest. Ophthalmol. Vis. Sci..

