# Supplementary material for: Automatic choroidal segmentation in OCT images using supervised deep learning methods
Source: Sci Rep. 2019 Sep 16;9:13298. doi: 10.1038/s41598-019-49816-4 (PMC6746702; doi:10.1038/s41598-019-49816-4)
Supplement: Supplementary file 1 — SUPPLEMENTARY INFO [file 41598_2019_49816_MOESM1_ESM.docx]

**Automatic choroidal segmentation in OCT images using supervised deep learning methods**

Jason Kugelman1, David Alonso-Caneiro1,2*, Scott A. Read1, Jared Hamwood1, Stephen J. Vincent1, Fred K. Chen 2,3,4 and Michael J. Collins1

1 Contact Lens and Visual Optics Laboratory, School of Optometry and Vision Science, Queensland University of Technology, Brisbane, Queensland, Australia

2 Centre for Ophthalmology and Visual Science, The University of Western Australia, Perth, Australia

3 Ocular Tissue Engineering Laboratory, Lions Eye Institute, Perth, Australia

4 Department of Ophthalmology, Royal Perth Hospital, Perth, Australia

[*d.alonsocaneiro@qut.edu.au](mailto:*d.alonsocaneiro@qut.edu.au)

| **Cifar CNN (CNN 1)** | | | | **Complex CNN (CNN 2)** | | | |
| --- | --- | --- | --- | --- | --- | --- | --- |
| **32x32** | **64x32** | **64x64** | **128x32** | **32x32** | **64x32** | **64x64** | **128x32** |
| 5x5 Conv. 32F (1,1)S (2,2,2,2)P | | | | 5x5 Conv. 32F (1,1)S (0,0,0,0)P | | | |
| 3x3 Max Pool. (2,2)S (0,1,0,1)P | | | | ReLU | | | |
| ReLU | | | | 3x3 Conv. 32F (1,1)S (0,0,0,0)P | | | |
| 5x5 Conv. 32F (1,1)S (0,0,0,0)P | | | | ReLU | | | |
| ReLU | | | | 3x3 Conv. 64F (1,1)S (0,0,0,0)P | | | |
| 3x3 Avg. Pool. (2,2)S (0,1,0,1)P | | | | 2x2 Max Pool. (2,2)S (1,1,1,1)P | | | |
| 5x5 Conv. 64F (1,1)S (0,0,0,0)P | | | | 3x3 Conv. 64F (1,1)S (0,0,0,0)P | | | |
| ReLU | | | | ReLU | | | |
| 3x3 Avg. Pool. (2,2)S (0,1,0,1)P | | | | 3x3 Conv. 128F (1,1)S (0,0,0,0)P | | | |
| 4x4 FC 64F | 8x4 FC 64F | 8x8 FC 64F | 16x4 FC 64F | 2x2 Max Pool. (2,2)S (1,1,1,1)P | | | |
| ReLU | | | | 5x5 FC  128F | 13x5 FC 128F | 13x13 FC 128F | 29x5 FC 128F |
| 1x1 FC 4F | | | | | | | |

Supplementary Table S1: CNN architectures used within this work. #F represents the number of filters, (#,#)S is the stride lengths (horizontal, vertical), and (#,#,#,#)P is the zero-padding applied to the input (top, bottom, left, right).

| **RNN** | | | |
| --- | --- | --- | --- |
| **32x32** | **64x32** | **64x64** | **128x32** |
| Vertical Bidirectional 16F (1,1)R | | | |
| Horizontal Bidirectional 16F (1,1)R | | | |
| Vertical Bidirectional 16F (2,2)R | | | |
| Horizontal Bidirectional 16F (2,2)R | | | |
| 8x8 FC 4F | 16x8 FC 4F | 16x16 FC 4F | 32x8 FC 4F |

Supplementary Table S2: RNN architecture used within this work. FC: fully-connected layer, #F: number of filters per pass, (#,#)R: receptive field size (height, width). 25% dropout added after each bidirectional layer.

| Year | Author/Reference | Key features | Network application |
| --- | --- | --- | --- |
| 2014 | Shelhamer, Long and Darrell^58^ | Basic upsampling layers, low-high skip connections | Standard |
| 2015 | Noh et al^59^ | Learnable deconvolutional network (decoder), encoder-decoder architecture | Standard |
| 2015 | Ronneberger, Fischer and Brox^60^ | U-Net, encoder-decoder skip connections, large number of decoder channels | Standard |
| 2016 | He et al^61^ | Residual learning | Residual |
| 2016 | He et al^62^ | Improved residual unit | Residual |
| 2016 | Drozdzal et al^63^ | Residual networks with decoder | Residual |
| 2018 | Zhang et al^64^ | U-Net with residual learning | Residual |
| 2016 | Visin et al^65^ | RNNs for semantic segmentation | RNN bottleneck |
| 2017 | Hu et al^66^ | Squeeze-excite block | Squeeze + excitation |
| 2018 | Roy et al^67^ | Squeeze-excite block variants (cSE, sSE, scSE) | Squeeze + excitation |
| 2018 | Roy et al^68^ | Squeeze-excite block in fully convolutional networks and optimal configuration | Squeeze + excitation |

Supplementary Table S3: Summary of previous work for semantic segmentation networks with key features from each identified along with the relevant network.

| **Method** | **Vitreous** | **Retina** | **Choroid** | **Sclera** |
| --- | --- | --- | --- | --- |
| **RNN (32x32)** | 99.83 (0.06) | **99.41** (0.12) | 96.97 (2.30) | 98.78 (1.14) |
| **RNN (32x32) [CE]** | **99.84** (0.06) | 99.37 (0.11) | 97.34 (1.30) | 98.99 (0.62) |
| **RNN (64x32)** | 99.83 (0.08) | 99.36 (0.13) | 97.33 (1.55) | 98.95 (0.78) |
| **RNN (64x32) [CE]** | 99.82 (**0.05**) | 99.36 (**0.10**) | 97.56 (**1.09**) | 99.05 (0.55) |
| **RNN (64x64)** | 99.82 (**0.05**) | 99.32 (0.11) | 97.55 (1.39) | 99.08 (0.67) |
| **RNN (64x64) [CE]** | 99.82 (0.06) | 99.37 (0.12) | **97.68** (1.18) | **99.12** (0.56) |
| **RNN (128x32)** | 99.81 (0.17) | 99.37 (0.20) | 97.42 (1.58) | 99.00 (0.76) |
| **RNN (128x32)** | 99.81 (0.12) | 99.36 (0.14) | 97.52 (1.26) | 99.04 (0.59) |
| **CNN 1 (32x32)** | 99.82 (0.14) | 99.38 (0.17) | 96.61 (2.54) | 98.63 (1.23) |
| **CNN 1 (32x32) [CE]** | 99.81 (0.06) | 99.33 (0.19) | 97.32 (1.37) | 98.98 (0.64) |
| **CNN 1 (64x32)** | 99.82 (0.06) | 99.33 (0.12) | 96.91 (2.45) | 98.77 (1.20) |
| **CNN 1 (64x32) [CE]** | 99.83 (**0.05**) | 99.32 (0.12) | 97.43 (1.12) | 99.02 (**0.53**) |
| **CNN 1 (64x64)** | 99.83 (**0.05**) | 99.40 (0.11) | 97.46 (1.89) | 99.00 (0.92) |
| **CNN 1 (64x64) [CE]** | 99.83 (**0.05**) | 99.33 (0.13) | 97.58 (1.54) | 99.08 (0.73) |
| **CNN 1 (128x32)** | 99.80 (0.06) | 99.32 (**0.10**) | 97.45 (1.43) | 99.01 (0.69) |
| **CNN 1 (128x32) [CE]** | 99.83 (0.06) | 99.35 (0.13) | 97.48 (1.11) | 99.06 (0.54) |
| **CNN 2 (32x32)** | 99.82 (0.22) | 99.32 (0.31) | 96.74 (2.14) | 98.70 (1.08) |
| **CNN 2 (32x32) [CE]** | 99.83 (**0.05**) | 99.38 (0.11) | 97.31 (1.44) | 98.96 (0.66) |
| **CNN 2 (64x32)** | 99.83 (0.06) | 99.34 (0.12) | 97.04 (1.90) | 98.84 (0.95) |
| **CNN 2 (64x32) [CE]** | 99.82 (**0.05**) | 99.19 (0.13) | 97.29 (1.25) | 99.02 (0.59) |
| **CNN 2 (64x64)** | 99.81 (0.06) | 99.30 (0.11) | 97.15 (2.05) | 98.87 (1.09) |
| **CNN 2 (64x64) [CE]** | 99.81 (**0.05**) | 99.24 (0.13) | 97.39 (1.45) | 99.03 (0.71) |
| **CNN 2 (128x32)** | 99.83 (0.06) | 99.33 (0.11) | 97.03 (1.89) | 98.85 (0.92) |
| **CNN 2 (128x32) [CE]** | 99.80 (0.12) | 99.29 (0.14) | 97.39 (1.31) | 99.01 (0.64) |
| **Baseline^37^** | 99.80 (0.19) | 98.87 (0.46) | 95.93 (3.81) | 98.40 (2.08) |

Supplementary Table S4: Dice overlap for each of the patch-based methods with comparison to the baseline. Mean percentage (%) and (per B-scan standard deviation) are reported for each of the four segmented regions. [CE] indicates that the network was trained and tested with images pre-processed using contrast enhancement. CNN 1: Cifar CNN, CNN 2: Complex CNN. The best result for each region is highlighted in bold text.

| **Method** | **Vitreous** | **Retina** | **Choroid** | **Sclera** |
| --- | --- | --- | --- | --- |
| **Standard** | **99.82** (0.06) | **99.41** (0.10) | 97.97 (1.06) | 99.21 (0.57) |
| **Standard [CE]** | **99.82** (0.06) | 99.40 (0.11) | 98.13 (0.91) | 99.29 (0.46) |
| **Residuals** | **99.82** (0.06) | **99.41** (0.10) | **98.15** (**0.81**) | 99.28 (0.44) |
| **Residuals [CE]** | **99.82** (0.06) | 99.38 (0.16) | 98.13 (0.92) | **99.30** (0.43) |
| **RNN** | 99.81 (0.06) | 99.35 (0.10) | 98.10 (1.02) | 99.29 (0.54) |
| **RNN [CE]** | **99.82** (0.06) | 99.39 (0.10) | 98.11 (1.06) | 99.28 (0.52) |
| **cSE** | 99.81 (0.20) | 99.40 (0.39) | 98.06 (1.06) | 99.24 (0.54) |
| **cSE [CE]** | **99.82** (0.06) | 99.38 (0.10) | 98.12 (0.90) | 99.29 (0.44) |
| **sSE** | **99.82** (**0.05**) | 99.40 (0.10) | 97.99 (0.96) | 99.22 (0.51) |
| **sSE [CE]** | **99.82** (0.08) | 99.37 (0.14) | 98.02 (1.01) | 99.25 (0.51) |
| **scSE** | 99.81 (0.12) | 99.38 (0.18) | 98.10 (0.95) | 99.27 (0.49) |
| **scSE [CE]** | **99.82** (0.06) | 99.39 (**0.09**) | 98.11 (0.85) | 99.28 (**0.42**) |
| **Combined** | **99.82** (0.06) | **99.41** (0.10) | 98.08 (1.12) | 99.25 (0.60) |
| **Combined [CE]** | **99.82** (**0.05**) | 99.39 (0.10) | 98.12 (0.88) | 99.29 (0.45) |
| **Baseline^37^** | 99.80 (0.19) | 98.87 (0.46) | 95.93 (3.81) | 98.40 (2.08) |

Supplementary Table S5: Dice overlap for each of the semantic segmentation methods with comparison to the baseline. Mean percentage and (per B-scan standard deviation) are reported for each of the four segmented regions. [CE] indicates that the network was trained and tested with images pre-processed using contrast enhancement. The best result for each region is highlighted in bold text.
